# Seagrass Canopy Photosynthetic Response Is a Function of Canopy Density and Light Environment: A Model for *Amphibolis griffithii*

**DOI:** 10.1371/journal.pone.0111454

**Published:** 2014-10-27

**Authors:** John D. Hedley, Kathryn McMahon, Peter Fearns

**Affiliations:** 1 Environmental Computer Science Ltd., Tiverton, Devon, United Kingdom; 2 School of Natural Sciences and Centre for Marine Ecosystems Research, Edith Cowan University, Joondalup, Western Australia; 3 Department of Imaging and Applied Physics, Curtin University of Technology, Perth, Western Australia; Auckland University of Technology, New Zealand

## Abstract

A three-dimensional computer model of canopies of the seagrass *Amphibolis griffithii* was used to investigate the consequences of variations in canopy structure and benthic light environment on leaf-level photosynthetic saturation state. The model was constructed using empirical data of plant morphometrics from a previously conducted shading experiment and validated well to *in-situ* data on light attenuation in canopies of different densities. Using published values of the leaf-level saturating irradiance for photosynthesis, results show that the interaction of canopy density and canopy-scale photosynthetic response is complex and non-linear, due to the combination of self-shading and the non-linearity of photosynthesis versus irradiance (P-I) curves near saturating irradiance. Therefore studies of light limitation in seagrasses should consider variation in canopy structure and density. Based on empirical work, we propose a number of possible measures for canopy scale photosynthetic response that can be plotted to yield isoclines in the space of canopy density and light environment. These plots can be used to interpret the significance of canopy changes induced as a response to decreases in the benthic light environment: in some cases canopy thinning can lead to an equivalent leaf level light environment, in others physiological changes may also be required but these alone may be inadequate for canopy survival. By providing insight to these processes the methods developed here could be a valuable management tool for seagrass conservation during dredging or other coastal developments.

## Introduction

Seagrass meadows are a dominant habitat of most coastal environments and provide important ecosystem services such as primary production, nutrient cycling, sediment stabilization, food and habitat for other organisms and trophic transfers to adjacent habitats [1]. Globally, these ecosystem services have been valued at an approximated US$ 19000 ha^−1^ yr^−1^ [2] but emerging understanding of the carbon storage capability of seagrass meadows implies this may be an underestimate [3]. Despite these recognized values, the area of seagrass is reducing world-wide at an increasing rate. Waycott et al. [4] estimated 29% of the known areal extent has disappeared since seagrass areas were initially recorded in 1879, and the rate of decline has accelerated in the last two decades.

The key anthropogenic pressures impacting seagrass meadows at local scales are urban, industrial and agricultural runoff, infrastructure development and dredging [5]. These pressures impact seagrasses directly via physical removal or indirectly through the introduction of pollutants such as nutrients, or suspended sediments that result in a reduction of light reaching seagrass meadows. Seagrasses are sensitive to light reduction as they are typically adapted to high light environments [1].

Increasing research is being undertaken to improve the management and conservation of seagrass meadows through improved understanding of the risks they face (e.g. [6]), developing bioindicators of the pressures they are exposed to [7] and thresholds of stressors such as light reduction which may differentiate sub-lethal effects from permanent loss of seagrass [8], [9]. In general, leaf-level photosynthetic activity in response to irradiance follows a ‘photosynthesis versus irradiance curve’, which is linear for subsaturating irradiances but becomes non-linear, as progressively increasing irradiance causes saturation of the photosynthetic electron transport chain, and finally attains a plateau phase, which is defined as maximal photosynthesis rate (*P*_max_) [10], [11]. A key physiological parameter that represents a species response to a given light level is *E*_k_, defined as the intersection between the initial linear slope and *P*_max_ on a P-I curve. *E*_k_ is frequently referred to as the ‘saturating irradiance’ [12], [13] although technically it is slightly below the irradiance at which full photosynthetic saturation occurs, and above the irradiance at which saturation starts to cause deviation from linearity. *E*_k_ can be empirically determined and for each species may vary over a restricted range due to physiological acclimatization or factors such as temperature [14].

Various light threshold analyses have been proposed as having predictive capability for seagrass mortality. Dependent on available data, light levels can be assessed with respect to different factors or components of the environment, including the water column light attenuation coefficients [15] or Secchi disk depths [16]; light at the top of the seagrass canopy expressed as percentage of surface irradiance [17], [18]; instantaneous or mean daily irradiance [8], [19] or the number of hours of irradiance above *E*_k_ per day, *H*_sat_, [8], [20]. These thresholds can also be integrated over time, which is relevant to management when pressures persist over particular durations, e.g., dredging or flood plumes. The percentage of days below a particular mean daily irradiance [8] or the sum of the hours of irradiance below *E*_k_ compared to reference conditions [9] are two examples for which thresholds have been proposed to predict the onset of seagrass mortality.

One important component that all of these thresholds do not consider is the interaction of the seagrass canopy itself with the benthic light field, since it is the amount of light reaching individual leaves of a seagrass that governs the plants photosynthetic response [21]. The photosynthetic activity in turn influences how the seagrass meadow responds to the changes in light [12] and overall plant productivity [22]. Canopy structure of seagrass meadows can also vary markedly due to natural variations in light [23] or in response to light perturbations [9]. Due to canopy self-shading, light levels at the top of the canopy may be very different to light levels within the canopy, and will vary throughout the canopy in a manner dependent on the incident benthic light field, canopy structure and bending angle of the leaves, which vary under water motion [24], [25], [26]. Therefore, a mechanistic explanation of how light levels affect canopy sustainability must include the interaction of the canopy structure with the incident light field.

In this study we developed a 3D model of a complex seagrass canopy (*Amphibolis griffithii*) of varying structure, from low to high leaf area index (LAI), by adapting the model described in [25] and [27]. We modeled the exposure of these virtual canopies to a number of environmentally relevant levels of light reduction to assess the amount of light reaching each leaf surface and how this varies under different canopy densities and positions due to movement associated with water motion. Finally, we assessed the canopy saturation state by relating the light each leaf receives to values of leaf-level *E*_k_ for *A. griffithii* found in the literature. The modeling scenarios were based on empirically quantified canopy structures from specific plant morphologies, and were designed to be comparable to a shading experiment that was conducted on *A. griffithii* in 2005 [9].

In summary, the objectives were:

- To develop a 3D canopy model for a seagrass species with a complex canopy, hence demonstrating an advance in technical capability with respect to the simple *Thalassia* morphology model of Hedley and Enríquez [25].
- To understand the consequences on within-canopy light capture and canopy saturation state of 1) canopy position: upright vs. moving under high wave action, 2) canopy structure: low to high LAI (1.27 to 7.65), and 3) light reduction: 0–95% shading
- To identify potential descriptors of canopy light levels which could have use for the management of seagrass beds under light reduction events such as dredging or coastal pollution.

## Methods

### Canopy structures

**Figure 1:**
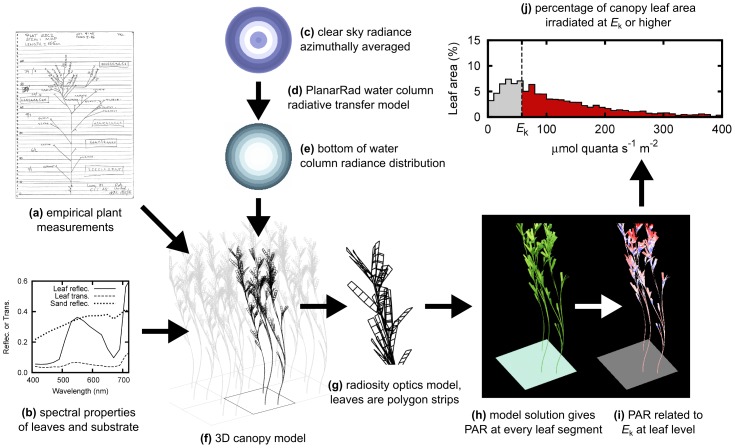
Overview of modelling system. (a, b) empirical data informs construction of 3D canopy model (f), (c, d, e) A plane-parallel model estimates directional radiance incident on the top of the canopy, (f, g, h) a geometric optical model handles radiative transfer to and between leaf segments, (i, j) PAR distribution over leaf area is reduced to the percentage of the canopy irradiated above leaf-level photosynthetic saturation, *E*_k_.

The modelling experiment was designed to mirror aspects of a previously published empirical shading manipulation experiment [9], [28]. The empirical study utilised an extensive (>6 ha) meadow of *Amphibolis griffithii* in 4.5 m water depth at Jurien Bay, Western Australia (30° 18′ 34″ S, 115° 00′ 26″ E; WGS84 datum). A control plus two-treatment shading experiment was conducted, the first phase of which ran from 10th March to 14th June 2005. Before and during the experiment individual *A. griffithii* plants were sampled and characterised in terms of stem and branch lengths, internodal distances, and number and dimensions of terminal leaves (Fig. 1a).

**Figure 2:**
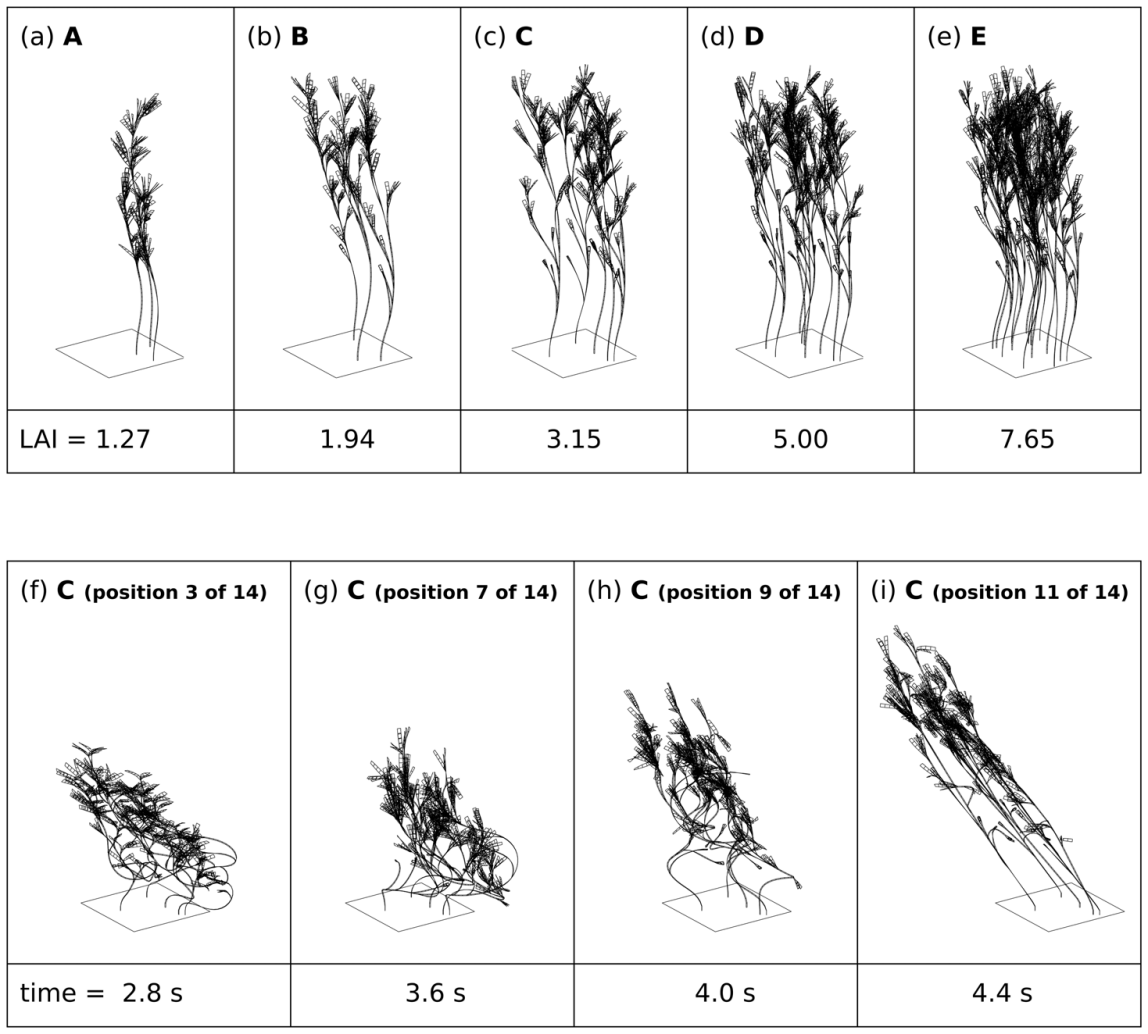
Example canopy structures and positions used in the model treatments. (a)–(e) low wave action canopy position for the five LAI treatments A to E. (f)–(i) subset of time sequence positions under high wave action for canopy C, all 14 positions were used in the optical model. In all cases the canopy structure is notionally repeated in all horizontal directions such that the square substrate section tessellates.

In the computer model, ten sets of individual plant data from the initial control sampling were replicated as vector mesh structures (Fig. 1f). The model plants were assembled into five canopies of leaf area index (LAI) from 1.27 to 7.65, by varying the choice and number of plants in a 20 cm×20 cm segment of substrate (Table 1, Fig. 2). The leaves and stems of the vector mesh structures were modelled as a point-mass and force system according to methods typically used for modelling cloth in the computer graphics industry [29]. A dynamic numerical integrator modelled the plant structures flexing naturalistically under a simple wave-action force model. Two wave actions, ‘high’ and ‘low’, were employed. In the dynamic model the low wave energy treatment plants were allowed to assume a typical upright position with no wave induced movement (Fig. 2). Under high wave energy plants underwent a vigorous cycle of forward and backward motion (Figs. 2f–i). From these dynamic models canopy structure treatments were extracted as instantaneous snapshots for each of the five LAI treatments: 1) a single snapshot for low wave action, 2) 14 snapshots through a cycle of movement for high wave action (Fig. 2). The 14 snapshots for high wave action were individually passed to the optical model (see below) and the results were averaged, thereby assuming the canopies undergo this movement continuously and photosynthetic response is the mean of the responses at any instant in time.

**Table 1:** Modelling experiment design.

LAI	Structure	Shading (%)	Hour
A −1.27	low wave energy (1 position)	0	×12
B −1.94	high wave energy (14 positions)	10	
C −3.15		25	
D −5.00		40	
E −7.65		50	
		60	
		70	
		85	
		95	

A fully-factored set of model runs were performed for each of five LAI treatments, 15 canopy structures and nine shading treatments over 12 hourly diurnal intervals, a total of 8100 runs.

### Water column optical model and shading

The canopy structures were input to the optical model for estimating diurnal leaf-incident irradiance. The model framework, previously described in [27] and [25], propagates sky radiance distributions through the canopy to give leaf incident irradiance in 17 wavebands of 20 nm width from 400–740 nm. Spectral irradiance can then be reduced to photosynthetically available radiation (PAR) at leaf level, and related to leaf tissue photosynthetic saturating irradiance, approximated by *E*_k_ (Fig. 1). To parameterise the model, hourly clear sky radiance distributions were produced using libRadtran and a directional radiance model [30], [31] corresponding to the Jurien Bay site on 27th April; the middle of the post summer 3 month trial phase in Lavery et al. [9] (Fig. 1c).

The sky radiance distributions were input to PlanarRad (http://www.planarrad.com), a plane parallel water column model functionally similar to HydroLight [32], [33] to estimate the hourly top of canopy radiance distribution (Fig. 1e). The model provides directional radiance tabulated over a hemisphere of zenith and azimuth angles, but to remove any dependency on sun azimuth and canopy orientation downwelling irradiances were azimuthally averaged to have only a zenith angle dependency (Fig. 1c, e). The water column utilised a library set of spectral inherent optical properties (IOPs, for details see [34]) which when input to the model produced a diffuse attenuation of planar PAR irradiance, *k*_d_, of approximately 0.2 m^−1^. In comparison, *k*_d_ values measured at the time of the empirical shading experiment ranged from 0.07 to 0.19 over a four month period but were 0.19 in April (Department of Parks and Wildlife, unpublished data). A set of nine modelled shading treatments were implemented by taking the top of canopy radiance distribution and reducing the values by 10%, 20%, etc. up to 95% (Table 1). Hence shading was spectrally neutral as was the shade cloth used in Lavery et al. [9], where the shading treatments were equivalent to 81–87% and 89–95% in our notation. The empirical study therefore represented quite a strong shading effect with respect to the modelled range. The water column optical model was additionally evaluated by comparing modelled top of canopy daily PAR irradiance against *in-situ* measurements from the associated study [28].

### Canopy structure optical model

The top of canopy irradiance was propagated through a geometrical optics model [25], [27] that accounts for inter-reflection and transmission between leaf segments. The spectral reflectance and transmittance of *A. griffithii* leaves was taken from the paper of Durako [35]. In this study we did not attempt to capture inter or intra-plant variability in leaf absorptance. This can be done [25], but the data collection requirements are onerous. All surfaces were considered Lambertian reflectors and transmitters. The underlying substrate reflectance was set from a library sand spectral reflectance that had a mean value of 0.33. The 20×20 cm modelled canopy segment was repeated periodically horizontally so the modelled canopy was of uniform LAI and has no edge (Fig. 1f).

Empirical measurements of PAR irradiance close to midday at both the canopy top and on the substrate underneath canopies of *A. griffithii* of differing LAIs were available for validation of the canopy optical model from the study of McMahon and Lavery [28]. Canopy transmission was measured in control and treatment plots of varying but known LAI through measuring the instantaneous photosynthetic photon flux density (PPFD, µmol m^−2^ s^−1^) at the top and base of the canopy. The light sensor (Odyssey PAR sensor) was calibrated against a standard calibration light source (Quartz Tungsten Halogen Reference Lamp operated at 3150°K from a LI-1800-02 Optical Radiation Calibrator). The low wave energy structure model treatments at the hour closest to midday were used to perform this validation, and a number of additional runs with different LAIs to those in Table 1 were added to further populate the validation data. An additional quality assurance protocol for the canopy optical model is to set the within-canopy water absorptance to zero and then verify energy conservation between the top of canopy incident and exitant irradiances and energy absorbed by all surfaces in the model [25]. This was performed for a subset of the runs in Table 1.

### Relation to photosynthetic properties

The model solution provided incident PAR at every point on every leaf at a resolution of approximately 0.5 cm^2^. This was then related to the leaf level saturation irradiance for *A. griffithii*, approximated by *E*_k_. Masini and Manning [12] evaluated *E*_k_ in *A. griffithii* as ranging from 25 to 55 µmol quanta m^−2^ s^−1^ for temperatures of 13°C to 23°C, of which the upper value is closer to the conditions of the empirical data from the associated study here. While Masini and Manning [12] did not assess physiological variation in *E*_k_ in *A. griffithii*, in the same study *Posidonia sinuosa* was shown to have *E*_k_ that varied from 50.4 to 39.1 µmol quanta m^−2^ s^−1^ in depths of 4 m and 12–15 m respectively. Therefore to accommodate a realistic variation in *E*_k_ in the absence of data, we have used the comparable range of 45–55 µmol quanta m^−2^ s^−1^ to put all of our results into the context of potential physiological variation in *E*_k_. To produce plots including the range and mid-point specific values of 45, 50 and 55 were used. Based on the value of *E*_k_ of 45 or 55 µmol quanta m^−2^ s^−1^, the model can report the instantaneous proportion of the leaf area of the canopy that is irradiated at or above the saturation irradiance. To interpret these results, for each canopy structure and shading treatment, a value termed *H* ^A^_sat_ was calculated as the time integral of the percentage leaf area above saturation in a 24 hour period, with units % leaf area×hour. This measure is discussed later, but was intended to be analogous to *H*_sat_, the daily top of canopy irradiated hours above saturation [9], [20] but also factoring in the canopy self-shading.

## Results

### Optical model validation

The sky radiance and water column model produced a daily top of canopy PAR dose of 11.0 mol quanta m^−2^, whereas the comparable *in-situ* measured average daily PAR irradiance over 3 months was 19.0 mol quanta m^−2^ [9]. Since our study was primarily concerned with the relative effect of the shading treatments and LAI this discrepancy is not of great importance, but could be due to: 1) the accuracy of the libRadtran sky radiance model (no validation data available); 2) the accuracy of the Odyssey PAR sensors, which can have issues in long-term stability (Slivkoff, pers. comm.), or; 3) the model water column *k*_d_(PAR), which was at the upper range compared to measurements taken during the empirical study (0.2 vs. 0.07 0.19). This deviation in *k*_d_(PAR) does provide an almost exact explanation for the discrepancy, but since *k*_d_(PAR) is a wavelength-integrated output of the model parameterised on spectral IOPs for absorption and backscatter it is not trivial to set an arbitrary value of *k*_d_(PAR). In the scope of this study, using the closest IOP set from actual measured data [34] was considered adequate. In reality the daily measured PAR was sometimes above and sometimes below the model value, so all things considered the modelled canopy PAR dose was reasonable and the discrepancy is inconsequential to the subsequent interpretation of the results.

**Figure 3:**
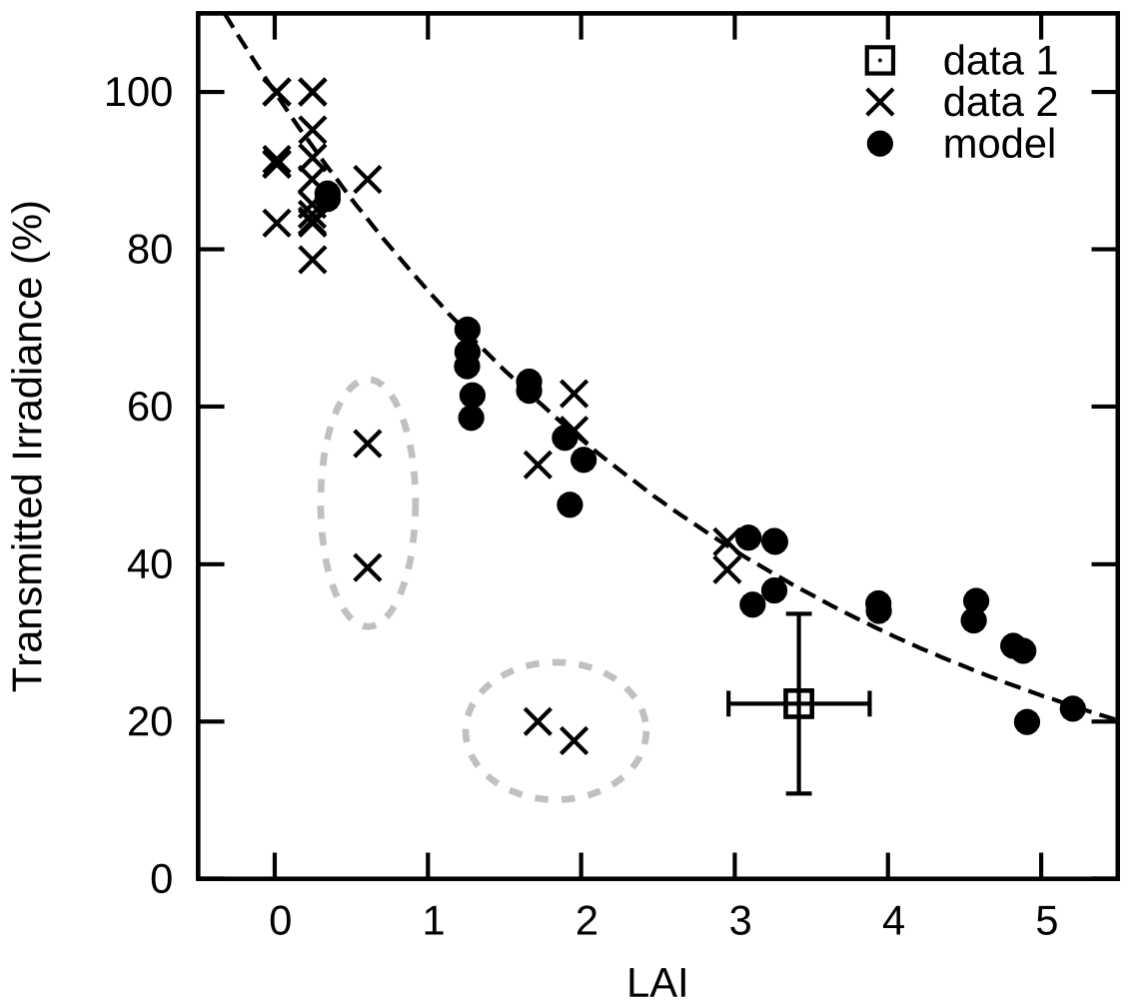
Percentage of downwelling top of canopy PAR irradiance reaching the substrate, as a function of LAI. Results for empirical in-situ measurements (data 1 and 2) and modelled estimates (model) are shown. Curve fit to model data points is *y* = 100 * exp(−0.29×LAI).×(data 2) - are 26 individual coincident measurements of LAI and irradiance above and below the canopy. ⊡ (data 1) is a single point based on a site mean of 13 LAI determinations from 2.84 to 4.09 and a set of associated but not spatially co-incident light measurements, error bars are one S.D. The four encircled points are the outliers referred to in the text. Transmittance data collection is described in McMahon and Lavery 2014 and corresponding LAI is unpublished data from Lavery et al. 2009. • are 25 model runs including both those described in detail and some additional runs. In the models runs solar zenith angle was approximately 28°.

The percentage of the incident top of canopy PAR irradiance transmitted to the substrate, as a function of leaf area index, validated well against empirical data (Fig. 3). The empirical data showed wide variation, but the modelled transmitted irradiances corresponded very closely to the upper bound of the empirical data. This is to be expected, since some of the real canopies contained free standing and epiphytic macroalgae which would have reduced the transmission beyond that described by the *A. griffithii* LAI alone. The upper bound points most likely represent the most monospecific *A. griffithii* canopies and correspond best to the model. An exponential function fit to the model data (*n* = 28) gave *r* ^2^ of 0.96, the fit of all 27 empirical data points to that same function gives an *r* ^2^ of 0.73. However, if only four outliers are removed (Fig. 3) the empirical data *r* ^2^ rises to 0.90. The model and empirical data therefore compare well, especially given the practical difficulty in making accurate within-canopy light measurements.

The performance of the model in terms of energy conservation was demonstrated in the subset of runs for which water absorption was set to zero. For the majority of runs energy losses were less than 2% and for all runs they were less than 3%. In practice, when water absorption is non-zero, energy conservation performance would be better than these figures suggest. The current model implementation requires water absorption to be set to zero for energy accounting, but this in itself removes a damping effect on the multiple scattering and increases energy losses through numerical errors. Therefore the model solutions for leaf incident irradiance can be considered, at worst, slight underestimates by around 2%.

### Effect of canopy structure and position on leaf level PAR

**Figure 4:**
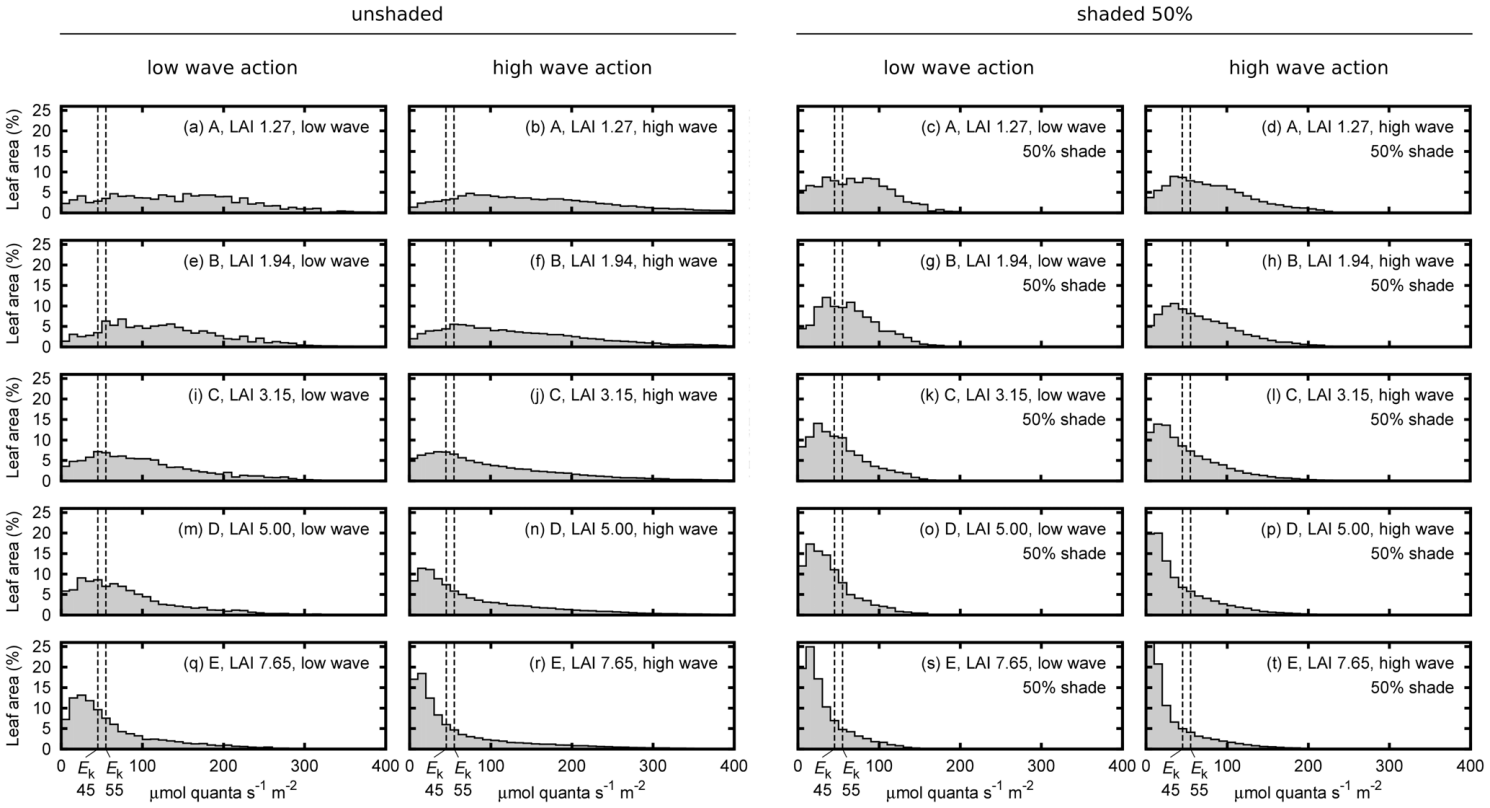
Distribution of leaf saturation state in the canopy in terms of percentage of leaf area at midday. Treatments are upright low wave action canopy positions and the average over the high wave action movement positions (left columns), and the same for 50% shading (right columns), for the five LAI treatments. The estimated photosynthetic saturation irradiances, *E*_k_, of 45 and 55 µmol quanta m^−2^ s^−1^ inferred from Masini and Manning (1997) are shown as vertical dotted lines.

As expected, the distribution of leaf level PAR irradiance became increasingly skewed to lower values as LAI increased (Figs. 4a, e, i, m, q). In low LAI canopies the distribution of PAR over leaf area was almost flat: leaf tissue received a wide range of PAR with almost equal probability, and much of it was above saturating irradiance at mid-day under the model conditions of clear sky and moderately clear water. In denser canopies the leaf level light distribution had a long high-end tail: many leaves received light below *E*_k_, but a few leaves received very high light (Fig. 4i). Overall the pattern was clearly linked to the relative openness or self-shading within the canopies. The range of *E*_k_ of 45 to 55 µmol quanta m^−2^ s^−1^ was generally small compared to range of irradiances the leaves experienced, but this was more true for the lower LAI and unshaded treatments (Fig. 4).

**Figure 5:**
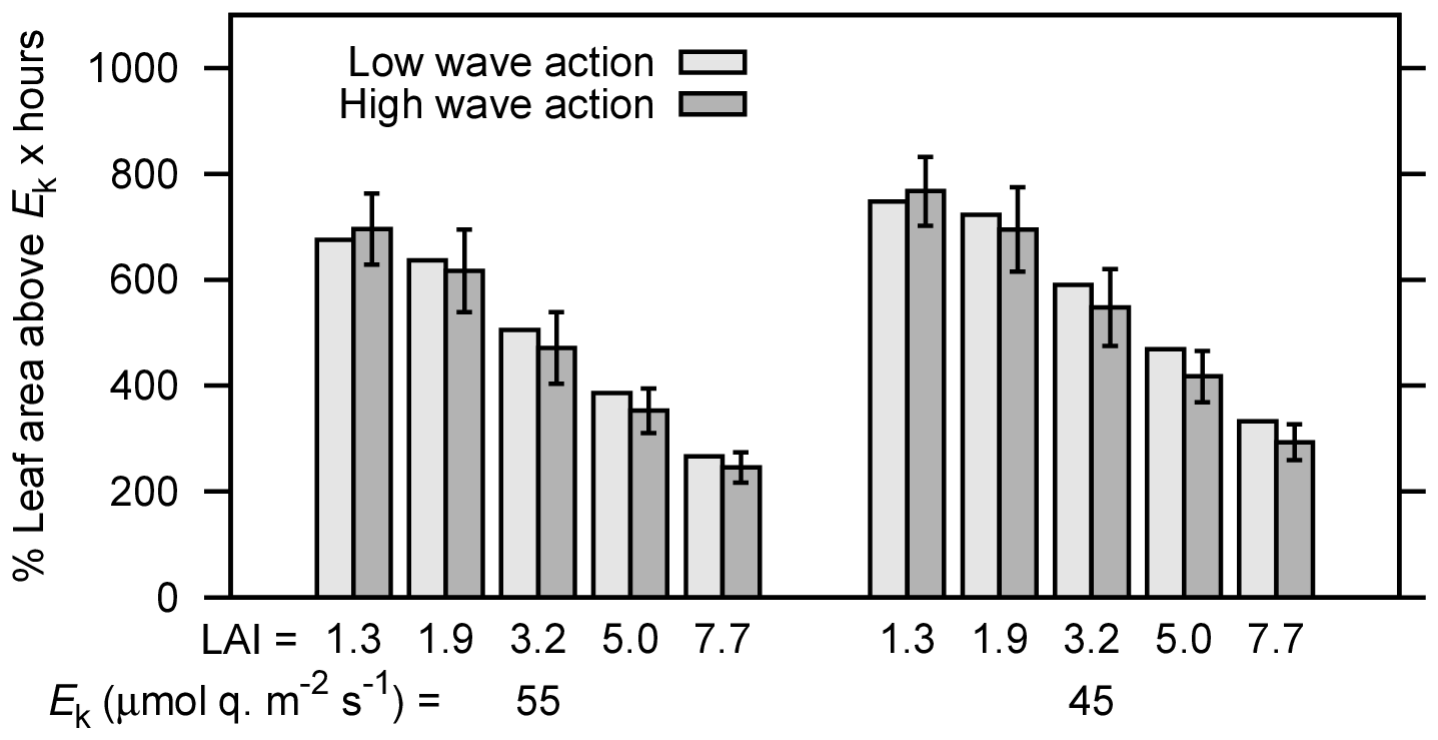
Time accumulated percentage of leaf area irradiated above photosynthetic saturation irradiance for low wave action and high wave action treatments, for *E*_k_ of 45 and 55 µmol quanta m^−2^ s^−1^. Error bars on high wave action treatment are the standard deviation over all 14 movement positions and hence indicate the range in the instantaneous canopy saturation state.

The treatments of canopy position of upright or moving under wave action appeared to have little effect on leaf level PAR irradiance (e.g. Fig. 4a vs. 4b). Numerically the canopy movement slightly reduced the daily integrated percentage of saturated leaf area for all but the lowest LAI (Fig. 5). However overall there was not a statistically significant difference at either *E*_k_ of 45 or 55 µmol quanta m^−2^ s^−1^ (paired value *t*-test, *p*>0.05). Therefore there is no evidence from our data that canopy movement affects time-integrated light capture. However the instantaneous light capture has a high variation under movement. While the standard deviation was 10–20% of the mean (Fig. 5) at some individual time points the saturated leaf area was up to 50% more or less than the mean. As expected, shading scaled the *x*-axis position of leaf-level irradiance distribution plots by the corresponding factor. That is, halving the top of canopy irradiance halved the leaf level incident irradiance at every leaf (Figs. 4b, d, f, h, etc.).

### Diurnally accumulated saturated leaf area

**Figure 6:**
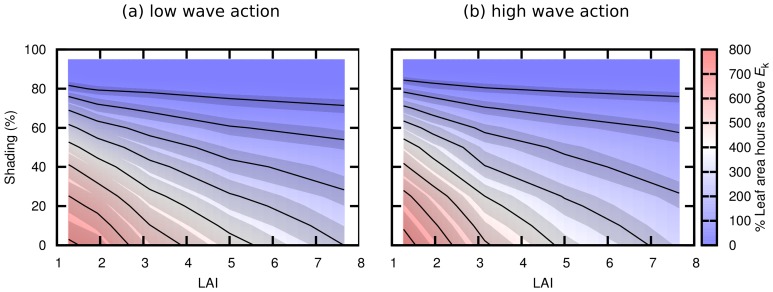
Time accumulated percentage of leaf area irradiated above photosynthetic saturation irradiance. The colour scale shows *H* ^A^_sat_, the “percentage leaf area hours” above *E*_k_, as a function of LAI and shading. (a) is for upright low wave action canopy structures, (b) is for the average over the high wave energy canopy positions. Contour lines are isoclines based on the mid value of *E*_k_ equal to 50 µmol quanta m^−2^ s^−1^ while the surrounding greyed region shows the limits for *E*_k_ of 45 to 55 µmol quanta m^−2^ s^−1^. The isoclines are located at *H* ^A^_sat_ of 50, 100, 200, and then steps of 100 up to 800% leaf area hours.

The accumulated percentage of leaf area above saturation over a twelve-hour day showed a complex relationship between both shading and LAI (Fig. 6). While, as expected, increasing either shading or LAI monotonically decreased the accumulated percentage of leaf area above saturation, the shape of the function was non-linear and there was an interaction between shading and LAI (Fig. 6a). The contour lines in Figure 6a make clear the trade-off between leaf area index and light with respect to the saturation state of the canopy. These lines show equal points in the LAI–shading function space, so for example a canopy of LAI 5.5 with no shading was equivalent to LAI 2.0 with 50% shading, with respect to the diurnally accumulated saturation of relative leaf area. The potential acclimation range of *E*_k_ from 45 to 55 µmol quanta m^−2^ s^−1^ (assuming water temperature at approx. 23°C) added a degree of freedom to the LAI-shading relationship approximately equivalent to 1 unit of LAI at low shading (e.g. along the *x*-axis of Fig. 6a), but this increased as shading increased to 60% or more (Fig. 6a). Therefore at low shading modifying *E*_k_ over the suggested range is equivalent to changing LAI by plus or minus one half.

For the high wave action treatment there is a small qualitative difference in the position of the contour lines in low LAI and low shading region as compared to the upright low wave action treatment (Fig. 6b vs. 6a). However a sensitivity analysis of the data tables underlying Figures 6a and 6b showed that these differences are equivalent to an error in the shading percentage of only 6 points or less. In other words, if in a practical application shading were quantified at discrete levels of 0, 5, 10, 15% etc. then the difference between upright and moving canopies would be negligible.

## Discussion

### Geometric optical modelling of seagrass canopies and validation

In terms of the geometrical optical modelling of seagrass canopies, the results presented here corroborate those of Hedley and Enríquez [25], showing that it is possible to construct a physical three dimensional model of a seagrass canopy and obtain acceptable validation against *in-situ* light measurements. Through-canopy transmission was estimated accurately for pure *Amphibolis* canopies, but the importance of considering epiphytes or other canopy constituents was underlined by the high variability of the empirical data, which in some cases had lower light penetration than the model predicted based on *Amphibolis* LAI alone.

With respect to morphological complexity, *A. griffithii* is on the more complex end of the spectrum in comparison to strap leaf morphologies of *Thalassia* and many other seagrass species [36], to which this modelling framework was previously confined. Therefore the potential for future models of other seagrass species is good as these two examples capture the range in canopy complexity. Hedley and Enríquez [25] used profiles of light through the canopy to derive a diffuse attenuation profile, *k*_d_, for validation. In this study only light measurements at the top and bottom of the canopy were available. However, this simpler validation may be preferable and adequate. In practice, empirical measurements of light profiles within canopies are difficult to make, and rarely fit well to exponential attenuation with depth. The measurements at the top and bottom of the canopy are the strongest “signal” for within-canopy attenuation and can be used to derive *k*_d_ if canopy height is known. So in future empirical work to which such modelling may be subsequently applied, we recommend measuring downwelling irradiance at the top and bottom of canopies, together with canopy height and LAI.

### Influence of shading, LAI and position on diurnal leaf photosynthetic saturation

The previous empirical shading study on *A. griffithii* [9] quantified the change in *H*_sat_ induced by shading, i.e. the total number of hours of top-of-canopy irradiance that was above photosynthetic saturation, as compared to the unshaded treatments. This quantity, summed over time, was demonstrated as a good indicator of changes in canopy biomass and capacity for subsequent recovery. However *H*_sat_ relates the top of canopy irradiance to leaf-level photosynthetic saturation irradiance and so ignores canopy self-shading and other structural factors, which therefore introduce an additional degree of freedom. Here, we factor in the canopy structure by considering the percentage of the canopy leaf area above saturating irradiance accumulated over time, *H* ^A^_sat_, with units of % leaf area×hour. This descriptor extends *H*_sat_ by reducing to a single number the interaction of the duration of saturating irradiance and the canopy self-shading. It can be roughly interpreted as the daily ratio of saturated photosynthesis to leaf area at canopy scale, and ranges from 0 to 1200 for plants completely saturated for 12 hours of daylight.

Considering the variation in *H* ^A^_sat_ with leaf area index and shading, as expected LAI has a strong effect on diurnal leaf saturation state (Fig. 6.). A change in LAI from 1 to 7 has as much effect as 60% shading (Fig. 6a), so the ambient light field cannot be treated independently from the canopy structure when photosynthetic processes at leaf level are of interest. Furthermore, the relationship between LAI and both shading and leaf saturation state is a non-linear interaction; Figure 6 represents a curved surface in both axes of shading and LAI. This occurs because while leaf level irradiance is a linear function of canopy level irradiance, the leaf level photosynthetic response is not a linear function of irradiance when the irradiance approaches or exceeds *E*_k_. As leaf level irradiance approaches and exceeds *E*_k_ the photosynthetic response levels off at *P*_max_. In general, since photosynthesis versus irradiance curves are non-linear in the region of the saturating irradiance any derived measure of leaf level photosynthetic activity will have a complex relationship with LAI unless all leaves are well below saturating irradiance.

Within this study there is no statistically significant evidence to support the statement that canopy movement effects light capture and photosynthetic response. Qualitatively it is interesting that under movement the lowest LAI canopy experienced an increase in daily saturation whereas the higher LAI canopies were systematically lower (Fig. 5). To test the statistical significance of this observation would require substantial further modelling effort and was outside the scope of this study, however at low LAIs sideways movement may serve to enhance light collection by spreading leaves out horizontally and making them insensitive to the directionality of incident light. Under wave action such flattening is intermittent and as we have shown here is not a factor of great photosynthetic significance. Additionally, under these conditions the optical consequences of surface waves and sediment resuspension should also be considered and may be more significant [37]. However, in other systems and species, canopy flattening can be a result of shallow water depth or tidal or estuarine flow [38]. In this case the semi-constant flattening of the canopy may be of optical significance.

### Potential for LAI modification as an acclimation response

Figure 6 indicates that modification of LAI is a possible response to maintain the saturation state of the canopy under reduced or enhanced light conditions. This role of morphological plasticity has been demonstrated in a number of experimental studies (e.g. [39]) and hypothesized as a regulatory mechanism in *Thalassia* [13]. From our model data (Fig. 6), if a canopy of LAI 7 is observed to reduce to LAI 4 after a period of 40% reduction in light, this loss of biomass might be interpreted as a trajectory of canopy decline but alternative interpretation is that of an acclimation response to maintain the leaf level photosynthetic state. This interpretation is independent of the mechanism by which it occurs. Leaf mortality might be considered just a by-product of inadequate light to maintain respiration, but if the net effect is a return to an unstressed leaf-level light regime then the distinction between a compensatory morphological adjustment and a decline is at best ambiguous. This argument is of course dependent on the definition of ‘decline’; it does not apply if net productivity per unit area rather than biomass is the criteria. Here, by ‘decline’ we mean an implied trajectory toward canopy eradication.

**Figure 7:**
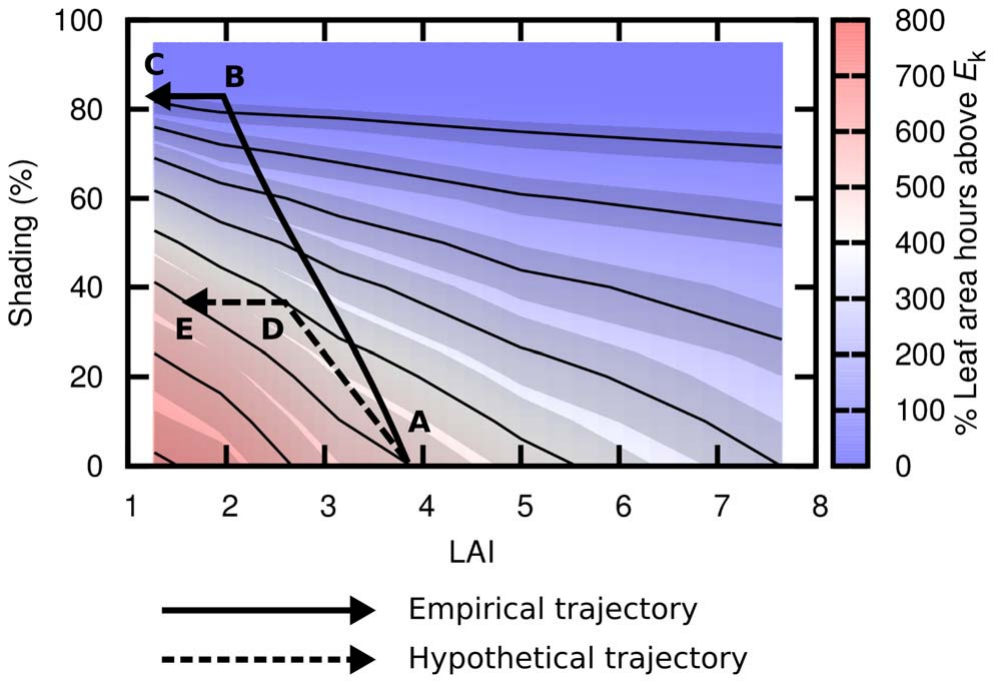
Trajectories of canopies from empirical shading experiment and hypothetical example. Underlying plot is as described in the caption of Figure 6a.

Under the interpretation of potential acclimation a key question is whether the reduction in LAI remains on the isocline for canopy saturation state (i.e. the contour lines in Fig. 6). A canopy that moves on a trajectory through LAI–shading ‘space’ such that it stays on a contour line is experiencing the same time integrated percentage of its leaf area at saturating irradiance (see A to E in Fig. 7). That is, it experiences the same daily photosynthetic saturation in relation to its leaf biomass. We might therefore hypothesise that if a canopy can sustainably exist at one point on an *H* ^A^_sat_ isocline, canopies can also sustainably exist at other points on that line, all other things being equal. Movement along an isocline can occur purely by modification of the LAI, alternative acclimation responses such as modification of *E*_k_ at the leaf-level will enable movement perpendicular to the isoclines, illustrated by the range around the isoclines in Figures 6 and 7 delimited by *E*_k_ of 45 to 55 µmol quanta m^−2^ s^−1^. Ignoring the latter possibility, and focussing on LAI modification alone, the prospects for long term survival of a canopy under a change in light environment can be estimated by following the isocline from its current location in LAI-shading space to the new light environment (*y*-axis) location. If at this location the LAI is greater than zero (judged by extrapolation), then the canopy could survive by thinning out to this LAI, at which point it will have the same relative photosynthetic saturation of its leaf area. In the following section we use this concept to interpret the results from the previous empirical shading experiment [9].

### Canopy trajectories in LAI and shading space

In the post-summer treatment of the shading experiment of Lavery et al. [9] canopies with an LAI of ∼4 had reduced to an LAI of ∼2 after three months of 84% shading. This change in LAI and shading can be represented as a trajectory on the *H* ^A^_sat_ map: Figure 7, point A to B. At 6 months of shading the LAI had reduced to one and by 9 months the canopy was almost eradicated and did not subsequently recover (Fig. 7, point C). Assuming for the moment that *H* ^A^_sat_, the accumulated percentage area of leaf saturation above *E*_k_, is a measure relevant to canopy sustainability then Figure 7 indicates that such a measure could have predictive power for canopy survival. In the previous section we postulated that canopies can move along the isoclines by modification of LAI alone. The initial reduction of LAI in the empirical data (Fig. 7, point A to B) occurred in the first three months in response to 84% shading. The trajectory cuts across the isoclines because there is a time lag as the canopy cannot become thinner instantaneously. At three months (Fig. 7, point B) the LAI has reduced to 2 but the shading is extreme so at the leaf level the light environment is still very much reduced. There can be two response pathways, either physiological changes may allow the canopy to exist on the new isocline, such as the mobilisation of stored reserves [40] or reductions in the saturating irradiance [23], or if such processes cannot bring about sufficient change then further LAI reduction is required in an attempt to return to an isocline closer to the original. In this case following the isocline to the right and extrapolating to the intercept with the *x*-axis it is clear that the light environment is equivalent to a canopy with huge LAI of at least 20+ in the original un-shaded situation. For the canopy to survive would require physiological changes that would permit canopies of these high LAIs to exist normally in this environment. Such canopies did not exist, hence physiological changes are insufficient (it is clear from Fig. 7 that variation in *E*_k_ is inadequate), hence the LAI continued to decrease and eventually the canopy was eradicated (Fig. 6, Point C).

The previous example is a straightforward case of severe light limitation, but with moderate shading (for example 40%, Fig. 7) the situation is more complicated. The empirical data of Lavery et al. [9] only contained shading at a minimum of 81% so the example of 40% in Figure 7 is hypothetical. If a situation of 40% shading is introduced, assuming the validity of *H* ^A^_sat_, it is clear that the canopy could survive by reducing LAI from 4 to 1 (moving along the isocline from point A to point E, Fig. 7). However, because of the time lag in reducing LAI, an initial trajectory in which LAI partially reduces (A to D, Fig. 7) is realistic and is likely to include physiological responses in tandem. For example *E*_k_ could decrease, but at LAI around 3 the range of *E*_k_ from 45 to 55 only allows accommodation of up to 20% shading at the most (Fig. 7). The existence of a time lag is supported by studies on different seagrass species that have incorporated less extreme shading treatments over short time-scales; physiological changes such as increases in chlorophyll and reduction in LAI occur after longer durations of reduced light [39]. At point D in Figure 7 the canopy lies on an isocline that represents a canopy of LAI 5.5 in the unshaded environment. If such canopies can sustainably exist, at the same depth, water clarity etc. then the canopy may survive at LAI of 2 to 3 (point D), rather than reducing LAI to 1 (point E). Either way, Figure 7 has predictive power for the canopy response in that if it is anticipated a 40% shading event may occur, e.g., from dredging activities, then it is clear that a canopy that is sustainable at LAI of 4 could reduce to LAI of 1, or, could induce physiological changes to maintain an LAI higher than 1, but in the latter case only if canopies greater than LAI of 4 currently exist in that environment.

The possible trajectories in LAI-shading space of Figure 7 are dependent on the capability and time constants of other physiological acclimation mechanisms. These mechanisms could include adjustments to photosystem kinetics to increase the efficiency (i.e. lower *E*_k_), increases in chlorophyll content and a:b ratio to enhance light capture, or mobilisation of stored carbohydrates for maintenance and growth of the existing leaf biomass [7]. To our knowledge there is no published data on photo-acclimation in *A. griffithii* under changing light conditions. However, unpublished data by co-author McMahon shows that under high levels of shading there are reductions in the saturating irradiance and other photo-acclimation responses, which maintain electron transport rates at unshaded values, but there is a time-limit over which this photo-acclimation is maintained of around 21 days. Therefore, the model as we have developed here is very relevant for predicting impacts associated with longer term reductions in light of over three weeks or more.

### Other measures of canopy scale photosynthetic response to light

In the previous discussion we have assumed that the concept of isoclines of equal light environment' with respect to *H* ^A^_sat_ is valid. Alternative measures may be more appropriate but this does not affect the primary concept that canopy self-shading can be equivalent to environmental shading, and that there are two mechanisms of photosynthetic acclimation: physiological and via canopy structure. Any alternative measure of the photosynthetically relevant light environment would likely have a similar form to that of Figures 6 and 7. The interaction of self-shading and the non-linearity of leaf-level photosynthesis must inevitably result in a complex canopy scale response to LAI and the light environment for all canopies that are subjected to irradiances above photosynthetic saturation. Another candidate measure would be the integration of photosynthesis over time, i.e. to propagate the leaf-level light through a photosynthesis versus irradiance (P-I) curve to give an integrated photosynthesis measure equivalent to µmol O_2_ evolution. In addition, plots of actual top of canopy PAR light levels may have greater descriptive power than percentage shading (Figures 6 and 7). Lavery et al. [9] observed different canopy changes at similar shading levels and interpreted these as being due to differences in the absolute light levels. In this study we suggested *H* ^A^_sat_ as a simple extension of the top-of canopy *H*_sat_, since that measure has been demonstrated to have predictive power for canopy sustainability [8], [9] and has been used in management contexts, and percentage shading was employed as a mirror of the empirical treatments. Clearly, there are many opportunities for further experiments and modelling to determine the most relevant measure of canopy photosynthetic response, the key point being that that measure needs to include within-canopy light propagation.

## Conclusions

Three dimensional canopy modelling of *Amphibolis griffithii* has revealed that the interaction of light levels and canopy density on canopy-scale photosynthetic activity is complex and non-linear, in particular due to the non-linearity of leaf-level photosynthesis at saturating irradiance. The accumulated percentage area of leaf saturation above saturating irradiance, *H* ^A^_sat_, was proposed as a measure relevant to canopy sustainability, based on extension of the equivalent top-of-canopy measure *H*_sat_ that has previously proved useful. The available empirical data were not sufficient to evaluate the efficacy of *H* ^A^_sat_ due to lack of lower shading treatments. Evaluating this measure and other candidates such as integrated leaf-level photosynthesis requires further experimental work. Nevertheless the principle has been demonstrated that plots of equal light environment' (Fig. 6) produced for different seagrass species, water depths, and water column optical properties could have practical management applications for predicting and interpreting canopy changes under light reduction events. Reduction in seagrass density in response to shading must be interpreted in terms of the leaf-level light environment. While physiological responses are also important, existing canopies in the same environment can provide information of the limits of physiological acclimation, and indicate if change in light levels will induce a trajectory to steady state sustainability, or to eradication. An important future step is to understand the time constants in change and recovery trajectories, to determine how long shading events can be tolerated and the required recovery periods. This information will be invaluable to coastal management.
